# Joint Angle-Doppler Estimation Algorithm Based on Time Reversal Post-Doppler Adaptive MUSIC in Low-Angle Multipath Environments

**DOI:** 10.3390/s20216186

**Published:** 2020-10-30

**Authors:** Chao Xiong, Chongyi Fan, Xiaotao Huang

**Affiliations:** College of Electronic Science and Technology, National University of Defense Technology, Changsha 410000, Hunan, China; chaoxiong@nudt.edu.cn (C.X.); xthuang@nudt.edu.cn (X.H.)

**Keywords:** joint estimation, multipath, multiple-input multiple-output (MIMO), post-Doppler adaptive MUSIC, time reversal (TR)

## Abstract

This letter proposes a time-reversal (TR) post-Doppler adaptive multiple signal classification (MUSIC) algorithm for multiple-input multiple-output (MIMO) radars, which addresses the joint estimation of angle and Doppler in diffuse multipath environments. First, an improving TR MIMO multipath model is proposed to avoid the ambiguity between the direction and Doppler in one round trip. Then, the letter designs a spatial filter matrix according to transmit-receive steering matrices, suppressing undesired round trips. Finally, we combine the post-Doppler adaptive MUSIC algorithm and the designed filter to estimate angle and Doppler jointly. Simulation results verify the applicability and effectiveness of the proposed model and algorithm.

## 1. Introduction

Low-angle tracking is a challenge in very high-frequency (VHF) radar array signal processing [1,2,3], which relates to two main difficulties. The first one is rich multipath in complex terrains, making it tough to distinguish the target signal from multipath signals by traditional methods. The other is that current low-angle multipath models ignore the effect of Doppler frequency. Consequently, the performance of detection probability and parameter estimation accuracy degrades significantly, especially for moving targets in low-angle environments. Multiple-input multiple-output (MIMO) radar exhibits excellent capabilities in parameter estimation by adapting the orthogonal waveform technique [4]. Therefore, applying MIMO radar in multipath scenarios has potential value.

To solve the first difficulty, conventional algorithms regard multipath as interference and suppress it [5]. Oppositely, the time-reversal (TR) technique, as a utilizing multipath methods, has been widely used in array signal processing [6,7,8,9,10,11,12,13]. As for TR MIMO parameter estimation, Tan proposes an adaptive TR MUSIC algorithm to accomplish direction of arrival (DOA) estimation in a mirror multipath scene by matching multipath channels [14]. Without referring to the precise multipath model in [14], Liu multiplexes the rows and columns and applies the forward-backward spatial smoothing technique to perform multiple targets’ parameter estimation [15]. However, these algorithms suffer from diffuse multipath which is difficult to be modeled in real low-angle scenes.

As a general method, joint estimation is useful to address the second difficulty caused by moving targets [16,17,18]. Nevertheless, there are few pieces of research about angle-Doppler estimation in low-angle environments. Taking diffuse multipath and Doppler frequency into consideration, Foroozan applies the traditional STAP algorithm to estimate the angle and Doppler frequency jointly in an urban multipath environment [19]. Under the hypothesis that each multipath has a different Doppler frequency, the algorithm has an ambiguity issue between the angle and Doppler in one round trip. Another way is to combine compressing sensing (CS) and TR to accomplish the joint estimation of DOA, the direction of departure (DOD), Doppler in an environment with abundant clutter [20,21]. The essential is to establish a complete CS data dictionary. However, it is not suitable in low-angle environments due to the uncertainty of diffuse multipath incident angles and Doppler frequencies.

Dealing with the difficulties in low-angle environments, the letter combines matrix filtering and post-Doppler adaptive MUSIC technique to estimate angle and Doppler frequency. First, it improves a TR MIMO multipath signal model to avoid the angle-Doppler ambiguity in round trips. Furthermore, to suppress undesired round trips causing by the combination of forwarding and backward scattering angles, the letter considers transmit-receive steering matrices and designs a corresponding spatial filter. It can suppress the paths in the stopband sector, passing the actual target’s round trip. Ultimately, a post-Doppler adaptive MUSIC algorithm is performed to realize the joint estimation in diffuse multipath scenes. Simulations in Section 4 verify the reasonableness and effectiveness of the proposed model and algorithm.

## 2. The Improved TR MIMO Multipath Signal Model

**Figure 1:**
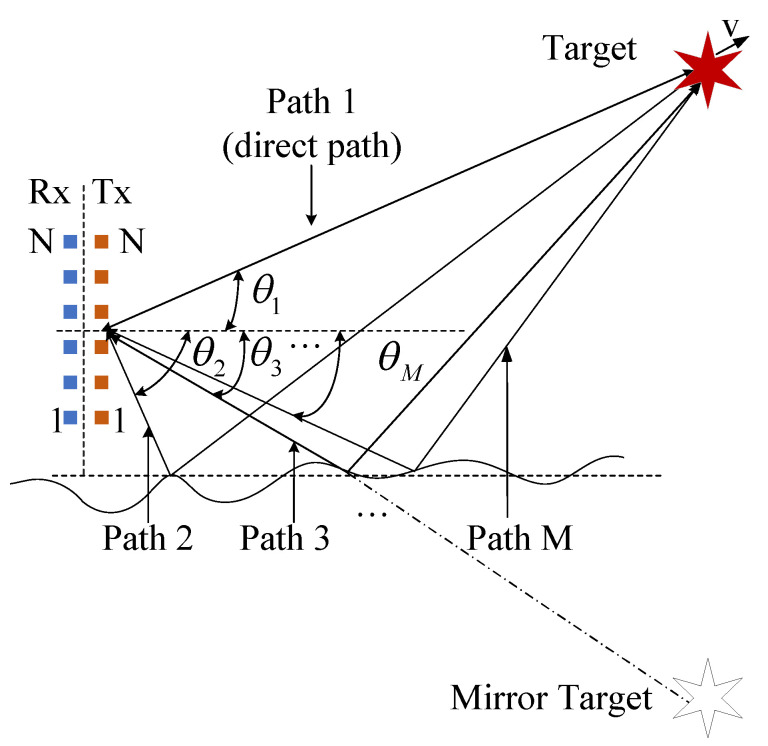
Schematic diagram of time reversal multiple-input multiple-output (TR MIMO) radar multipath model.

As shown in Figure 1, consider a monostatic MIMO radar, which is composed of a uniform linear array with *N* antennas separated by a half wavelength at both the transmitter and receiver. The transmission paths include one direct-path and M−1 reflection paths for general consideration. The attenuation factor, delay and arrival angle of *i*-th path are $\alpha_{i}$, $\tau_{i}$ and $\theta_{i}$, respectively. Besides, the direct-path angle is above 0° while other angles are below 0°.

### 2.1. MIMO Radar Signal Model

The first probing signal emitted by each transmit element is $f_{n}\left(t\right)e^{j2\pi f_{c}t}\left(1\leq n\leq N\right)$, where $f_{c}$ is the carrier frequency, $f_{n}\left(t\right)$ is the baseband envelope of the probing signal. All *N* transmitting signals can be written in the vector form: $f={\left[f_{1},f_{2},\ldots ,f_{N}\right]}^{T}$. Suppose that the baseband signal is orthogonal to each other, i.e., $ff^{H}=I_{N}$. The receiving signal of *j*-th (1≤j≤N) element is [19]:

(1) $$\begin{array}{cc} r_{j}\left(t\right) & =\sum_{m_{b}=1}^{M}\sum_{m_{f}=1}^{M}\sum_{n=1}^{N}\overset{\alpha_{l}}{\overset{︷}{\alpha_{m_{f}}\alpha_{m_{b}}}}f_{n}\left(t-\tau_{nm_{f}}\left(t\right)-\tau_{jm_{b}}\left(t\right)\right)\times e^{j\omega_{c}\left(t-\tau_{nm_{f}}\left(t\right)-\tau_{jm_{b}}\left(t\right)\right)}+n_{j}\left(t\right), \end{array}$$

where $m_{b}$ and $m_{f}$ represent the backward scattering path and forward scattering path, $l=(m_{f},m_{b})$ is a round trip. $\tau_{nm_{f}}$ and $\tau_{jm_{b}}$ are the propagation delays via forward and backward multipath between the *n*-th transmit element to the target and from the target to the *j*-th receive element, respectively. The carrier angular frequency $\omega_{c}=2\pi f_{c}$, and $n_{j}\left(t\right)$ is the observation noise of the *j*-th element.

Denote $A\left(\Theta_{l}\right)=\alpha_{R}\left(\theta_{m_{b}}\right)\alpha_{T}^{T}\left(\theta_{m_{f}}\right)$ as the transmit-receive steering matrix, where $\alpha_{R}$ and $\alpha_{T}$ are the steering vectors of a uniform linear array. They can be described as:

(2) $$\begin{array}{c} \alpha_{R}\left(\theta_{m_{b}}\right)={\left[1,e^{-j\omega_{c}\tau_{1}^{T}\left(\theta_{m_{b}}\right)},\ldots ,e^{-j\omega_{c}\tau_{N}^{T}\left(\theta_{m_{b}}\right)}\right]}^{T}, \end{array}$$

(3) $$\begin{array}{c} \alpha_{T}\left(\theta_{m_{f}}\right)={\left[1,e^{-j\omega_{c}\tau_{1}^{T}\left(\theta_{m_{f}}\right)},\ldots ,e^{-j\omega_{c}\tau_{N}^{T}\left(\theta_{m_{f}}\right)}\right]}^{T}. \end{array}$$

To simplify the expression, we rewrite the received signal in the vector form, $r={\left[r_{1},r_{2},\ldots ,r_{N}\right]}^{T}$ is recorded as:

(4) $$\begin{array}{c} r\left(t\right)=\sum_{l=1}^{L}\overset{{\tilde{\alpha }}_{l}}{\overset{︷}{\alpha_{l}e^{-j\omega_{c}\tau_{l}\left(0\right)}}}e^{-j\omega_{D_{l}}t}A\left(\Theta_{l}\right)f\left(t-\tau_{l}\left(0\right)\right)+n\left(t\right), \end{array}$$

where n(t) is the observation noise in MIMO radar.

### 2.2. TR MIMO Radar Signal Model

According to the principle of TR, r(t) is time-reversed, conjugated, energy normalized by *c* and retransmitted. The second transmitting signal is $cr^{*}\left(-t\right)$, where ${\left(\cdot \right)}^{*}$ represents the conjugation. The normalization coefficient *c* is $c=\sqrt{{\left||f|\right|}_{2}/{\left||r|\right|}_{2}}$.

With the equal amount of transmitting elements and receive elements, the second receiving signal x(t) can be written as:

(5) $$\begin{array}{cc} x(t) & =c\sum_{l^{\prime }=1}^{L}{\tilde{\alpha }}_{l^{\prime }}e^{-j2\omega_{D_{l}}t}A(\Theta_{l^{\prime }})r^{*}(-t+\tau_{l^{\prime }\left(0\right)})+v\left(t\right)\\ \; & \approx c\sum_{l=1}^{L}{\left|{\tilde{\alpha }}_{l}\right|}^{2}e^{-j2\omega_{D_{l}}t}\overset{A_{\mathrm{TR}}}{\overset{︷}{A\left(\Theta_{l}\right)A^{*}\left(\Theta_{l}\right)}}f^{*}\left(-t\right)+w\left(t\right), \end{array}$$

where v(t) is the observation noise in TR MIMO radar, while w(t) is the accumulated noise, which takes n(t) and v(t) into account. The approximation in Equation (5) is vaild due to the super-resolution focusing property of TR [13].

The signal model in (5) is described as the new model, which is different from the model in reference [19](original model). The difference reflects in the transmit-receive steering matrix $A_{\mathrm{TR}}$. Specifically, the steering matrix in the original model is $A^{T}\left(\Theta_{l}\right)A^{*}\left(\Theta_{l}\right)$, while our steering matrix is $A\left(\Theta_{l}\right)A^{*}\left(\Theta_{l}\right)$. It satisfies the principle of matrix multiplication due to the suppose that the number of elements in the receiving and transmitting arrays is equal.

In the post-Doppler adaptive beamforming framework, assume the second received signals consist of *K* pulses with a constant pulse repetition interval (PRI) in one coherent processing interval (CPI) and the *k*-th (0≤k≤K−1) pulse’s signal can be written as:

(6) $$\begin{array}{c} x_{k}\left(t\right)=c_{k}\sum_{l=1}^{L}{\left|{\tilde{\alpha }}_{l}\right|}^{2}e^{-j2\omega_{D_{l}}\left(\tau_{l}\left(0\right)+kTr\right)}\overset{A_{\mathrm{TR}}}{\overset{︷}{A\left(\Theta_{l}\right)A^{*}\left(\Theta_{l}\right)}}f^{*}\left(-t\right)+w_{k}\left(t\right), \end{array}$$

where Tr is the PRI, $c_{k}$ is the *k*-th pulse’s normalization coefficient. As the reference [22] does, we suppose that $c_{k}=c$ for all the pulses.

Applying matched filtering to the received signal in Equation (6), the new signals $y_{k}\left(t\right)$ are given by [15]:

(7) $$\begin{array}{cc} y_{k}\left(t\right) & =E[x_{k}\left(t\right)f^{T}\left(-t\right)]\\ \; & =c\sum_{l=1}^{L}{\left|{\tilde{\alpha }}_{l}\right|}^{2}e^{-j2\omega_{f_{l}}\left(\tau_{l}\left(0\right)+kTr\right)}\overset{A_{\mathrm{TR}}\left(\Theta_{l}\right)}{\overset{︷}{A\left(\Theta_{l}\right)A^{*}\left(\Theta_{l}\right)}}+u_{k}\left(t\right), \end{array}$$

where $u_{k}\left(t\right)=E\left[w_{k}\left(t\right)f^{T}\left(-t\right)\right]$ is a new noise matrix whose elements obey the Gaussian distribution.

## 3. The Post-Doppler Adaptive MUSIC Algorithm

This section first briefly describes the angle-Doppler ambiguity problem, then introduces the proposed post-Doppler adaptive MUSIC algorithm from two steps.

Consider two different round trips with the indexes $l=(\theta_{m_{f}},\theta_{m_{b}},\omega_{D_{f}},\omega_{D_{b}})$ and $l^{\prime }=\left(\theta_{m_{f}},\theta_{m_{b^{\prime }}},\omega_{D_{f}},\omega_{D_{b^{\prime }}}\right)$. The forward scattering angles are same and recorded as $\theta_{m_{f}}$, while the two backward scattering angles are different and described as $\theta_{m_{b}}$, $\theta_{m_{b^{\prime }}}$, respectively. The corresponding forward Doppler velocities are the same and recorded as $\omega_{D_{f}}$, the backward Doppler velocities are different and denoted as $\omega_{D_{b}}$ and $\omega_{D_{b^{\prime }}}$, respectively. Using the original model, the two transmit-receive matrices $A_{{\mathrm{TR}}_{l}}=A_{{\mathrm{TR}}_{l^{\prime }}}$, but $\omega_{l}=\left(\omega_{D_{f}}+\omega_{D_{b}}\right)\neq \left(\omega_{D_{f}}+\omega_{D_{b^{\prime }}}\right)=\omega_{l^{\prime }}$. It means that for two paths, as long as they have the same forward scattering angle even with different backward scattering angles, they can obtain the same transmit-receive steering matrix. Unfortunately, the Doppler velocities of the two paths are generally different. This is precisely the angle-Doppler ambiguity problem brought about by the reference [19]. However, it is a one-to-one correspondence between the matrix $A_{{\mathrm{TR}}_{l}}$ and the Doppler velocity $\omega_{l}$ in the new model according to (6). Compared with the original model, the new model introduces redundant angle-Doppler combinations while avoiding the angle-Doppler ambiguity problem. These redundant combinations affect the accuracy of parameter estimation. Therefore, the following shows how the algorithm suppresses undesirable multipath signals.

### 3.1. Design of the Matrix Spatial Filter

The essential of the conventional matrix spatial filter is to constrain the array response of the stopband and passband sectors. Correspondingly, the filter G has the following characteristics [23]:

(8) $$\begin{array}{c} G^{H}a\left(\theta \right)=\left\{\begin{array}{cc} a(\theta ), & \theta \in \Theta_{p}\\ \;\;0, & \theta \in \Theta_{s} \end{array}\right., \end{array}$$

where a(θ) is the steering vector of the angle θ in a uniform linearly array, while $\Theta_{p}$ and $\Theta_{s}$ represent the passband sector and the stopband sector, respectively.

In terms of the TR MIMO radar system, the passband sector refers to the round trips where the backscatter angles and the forward scattering angles are greater than 0°. The other situations are included in the stopband sector. By using the least-squares passband criterion of the peak stopband constraint, the designed spatial filter matrix G can be expressed as:

(9) $$\begin{array}{c} \min_{G}\;\sum_{j=1}^{Np}{∥G^{H}b\left(\Theta_{l_{j}}\right)-b\left(\Theta_{l_{j}}\right)∥}^{2}\\ s.t\;∥G^{H}b\left(\Theta_{l_{i}}\right)∥\leq \varepsilon ,\Theta_{l_{i}}\in \Theta_{s},i=1,2,.\ldots Ns\\ \;\;\;{∥G^{H}∥}_{F}\leq \delta , \end{array}$$

where G is the spatial filter, $b\left(\Theta_{l}\right)=\mathrm{vec}\left(A_{\mathrm{TR}}(\Theta_{l}\right))$ and vec(·) denotes the vectorization operation. $\Theta_{l_{j}}$ and $\Theta_{l_{i}}$ substitute the parameters of *j*-th round-trip in the passband and the parameters of *i*-th round-trip path in the stopband, respectively. ||·|| represents the Frobenius norm, ε is the corresponding stopband attenuation, ${∥G^{H}∥}_{F}\leq \delta$ constrains the noise power at the output of the designed filter.

Let $g={\left(\mathrm{vec}\left(G\right)\right)}^{*}$, the optimization problem in Equation (9) can be replced by the equal second order cone programming (SOCP) form:

(10) $$\begin{array}{c} \min_{g}\;\sum_{j=1}^{Np}∥\left[I⊗B^{T}\left(\Theta_{p}\right)\right]g-\mathrm{vec}\left[B^{T}\left(\Theta_{p}\right)\right]∥\\ s.t\;∥I⊗b^{T}\left(\Theta_{l_{i}}\right)∥\leq \varepsilon ,\Theta_{l_{i}}\in \Theta_{s},i=1,2,\ldots ,Ns\\ \;\;\;∥g∥\leq \delta , \end{array}$$

where I denotes the identity matrix, ⊗ is the Kronecker product. $B\left(\Theta_{p}\right)=\left[b\left(\Theta_{l_{j=1}}\right),b\left(\Theta_{l_{j=2}}\right),\ldots ,b\left(\Theta_{l_{j=N_{p}}}\right)\right]$.

### 3.2. Post-Doppler Adaptive MUSIC Processing

Applying matrix spatial filtering to *k*-th pulse’s receiving signal, the new signal $z_{k}$ is:

(11) $$\begin{array}{c} z_{k}=G^{H}\mathrm{vec}\left[y_{k}\right],\;k=1,2,\ldots ,K. \end{array}$$

Stacking all *K* pulses’ data in the vector form, $Z={\left[z_{1},z_{2},\ldots ,z_{K}\right]}^{T}$ is described as:

(12) $$\begin{array}{c} Z=\sum_{l=1}^{L}{\left|{\tilde{\alpha }}_{l}\right|}^{2}q_{l}+U, \end{array}$$

where $q_{l}$ is the new space-time steering vector. $q_{l}=a_{f}\left(l\right)⊗(G^{H}b(\Theta_{l}))$ and $a_{f}\left(l\right)=c{\left[1,e^{j2\omega_{f_{l}}Tr},\ldots ,e^{j2\omega_{f_{l}}\left(K-1\right)Tr}\right]}^{T}$. U represents the vectorized noise matrix of all *K* pulses.

The angle-Doppler MUSIC spectrum P(l) is:

(13) $$\begin{array}{c} P\left(l\right)=\frac{d_{l}^{H}d_{l}}{d_{l}^{H}E_{n}E_{n}^{H}d_{l}}, \end{array}$$

where $d_{l}$ denotes the space-time steering vector of path *l*, and its structure is similar to $q_{l}$. For *l*-th path, the forward scatering angle $\theta_{m_{f}}$ and the backward scatering angle $\theta_{m_{b}}$ are equal and greater than 0°. $R_{Z}$ represents the covariance matrix of Z, $E_{n}$ is the noise subspace acquired by the eigen-decomposition of $R_{Z}$, which contains all the eigenvectors that corresponding to the smallest NNK−1 eigenvalues.

## 4. Simulation Results

This section verifies the capability of the proposed algorithm (new method) from four aspects and demonstrates the performance by comparing it with the conventional STAP algorithm in [19] (original method). The basic parameters in experiments were as follows: array elements number N=12, path number M=4, carrier frequency $f_{c}=200$ MHz, and there were 256 snapshots.

### 4.1. Magnitude Response of the Matrix Spatial Filter

This simulation designed a matrix spatial filter used for the TR MIMO radar. Suppose both the backward scattering angle $\theta_{m_{b}}$ and the forward scattering angle $\theta_{m_{f}}$ of all the round-trips ranged from $-20^{{}^{\circ}}$ to 5° with 1° interval. The magnitude response of the stopband sector was below −40dB (ε=1.44), while the passband response remained steady as much as possible.

**Figure 2:**
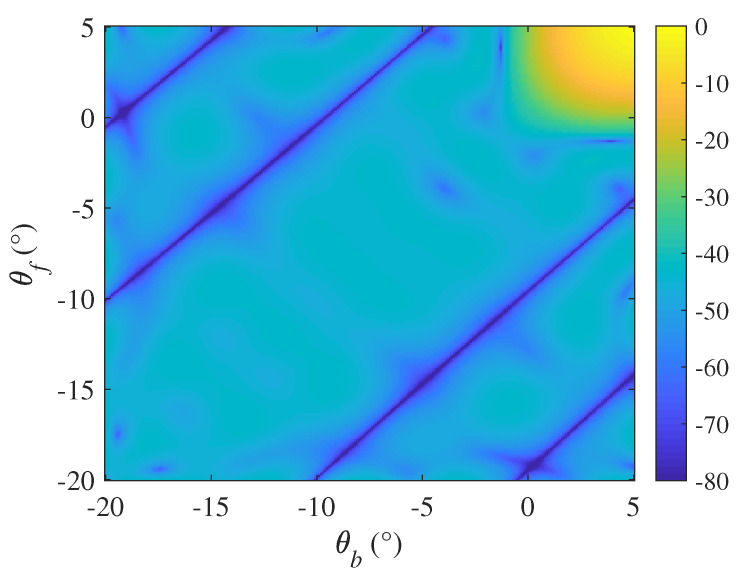
Magnitude response of the designed matrix spatial filter ($\theta_{b}$ and $\theta_{f}$ represent the backward multipath angle and forward multipath angle respectively).

As shown in Figure 2, the filter output of the stopband sector is strictly below −40dB, while the distortion within the passband sector was relatively small. This result shows that the designed filter was able to suppress the signal generated by multipath and had a relatively small impact on the direct wave signal of the real target.

### 4.2. Root Mean Square Errors (RMSEs) Versus SNRs

This experiment examines the applicablity of the proposed algorithm. DOAs of each path are $2^{{}^{\circ}},-2^{{}^{\circ}},-8^{{}^{\circ}},-20^{{}^{\circ}}$, respectively, corresponding delays were 0ns,3ns,8ns,15ns. The normalized Doppler frequencies are 0.2,0.16,0.1,0.06 (normalized by $\frac{1}{Tr}$), while the attenuation factors were randomly set as: 1,0.8730−0.0302i,0.4541+0.3012i,−0.5104+0.0915i. In whole simulations, SNR varied from −10dB to 10dB uniformly with 5dB interval and 1000 Monte Carlo trials were executed at each SNR.

**Figure 3:**
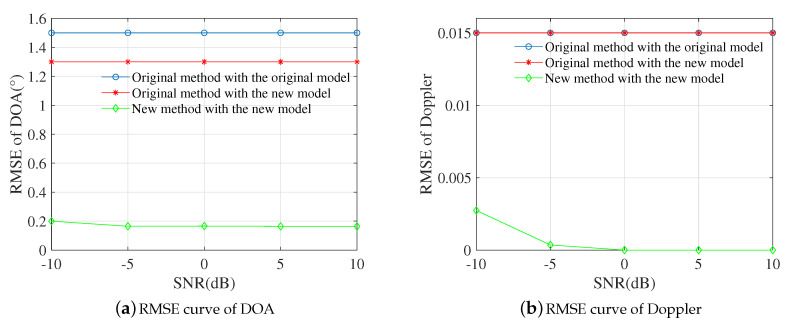
Root mean square error (RMSE) curves of DOA and Doppler versus SNRs.

As shown in Figure 3a, the RMSE of DOA obtained by the original method was unchanged with the improvement of SNRs neither under the original model nor the new model, which was similar to the normalized Doppler in Figure 3b. However, the RMSE under the new model is less than that under the original model, which seems the new model may increase estimation accuracy because of avoiding ambiguity. Nevertheless, parameter accuracy is still to be improved. Compared with the original method, the RMSE of DOA obtained by the new algorithm was below $0.2^{{}^{\circ}}$ at SNR = −10dB and decreased to a constant value with the increase of SNRs eventually, which was the same as the Doppler. The RMSE of the proposed algorithm was obviously lower than the original method, which benefited from the suppression of undesired round trips.

### 4.3. RMSE Distributions

This experiment verified the proposed algorithm’s applicability in different multipath scenes. The arrival angle, attenuation factor, delay and normalized Doppler frequency of the direct-path were 2°, 1, 0ns and 0.2, respectively. There was a mirror path and two diffuse multipath, while the parameters were randomly generated. We simulated 100 different multipath scenes with 100 Monte Carlo trials for each scene at SNR = 5 dB.

**Figure 4:**
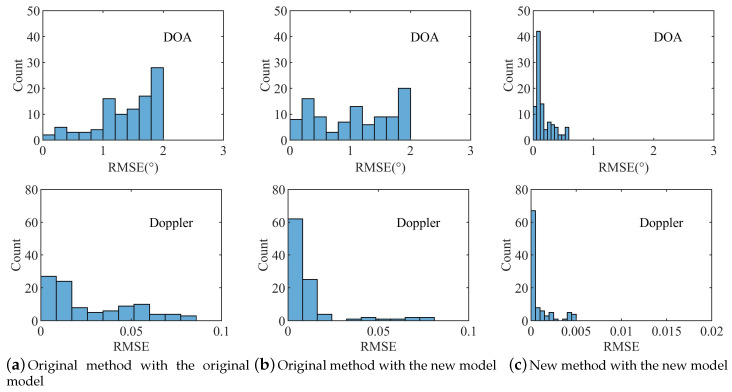
RMSE histograms of three cases (the top subplots are RMSE histograms of DOA, while the bottom subplots are RMSE histograms of Doppler).

Figure 4 plots the RMSE histograms of 100 different multipath environments in three situations. Compared with the other two cases from the aspects of the RMSE span range, our algorithm had an excellent performance in both DOA and Doppler. The result shows that our algorithm was more suitable in different multipath scenes.

### 4.4. RMSE of Different DOAs at A Fixed SNR

This experiment verified the applicability for different direct-path incident angles. For each direct-path angle, there was a mirror angle and two randomly generated angles between $-5^{{}^{\circ}}$ and $-20^{{}^{\circ}}$. The direct-path angle ranged from $1.1^{{}^{\circ}}$ to 2° with the step angle of $0.1^{{}^{\circ}}$, while all the normalized Doppler frequencies were randomly generated between 0 and 0.2. The SNR was 5dB, and 500 Monte Carlo trials were performed for each direct-path angle.

**Figure 5:**
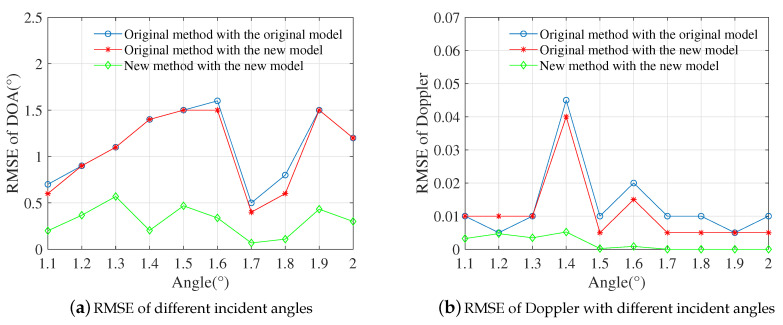
RMSE of three cases at SNR = 5dB.

Figure 5 describes the performance of the proposed joint estimation algorithm for different incident angles. For each direct-path angle, the RMSE of the proposed algorithm was lower than the corresponding RMSE of the conventional method. Moreover, the fluctuation of our RMSE was smaller than that of traditional STAP. Due to the randomness of parameters, the RMSEs were generally different even with the same SNR. The result further verified the superiority of the proposed algorithm.

## 5. Conclusion

In this letter, a post-Doppler adaptive MUSIC algorithm used for angle-Doppler joint estimation in diffuse multipath environments is proposed. The novel algorithm applies the matrix spatial filtering technique and post-Doppler adaptive MUSIC algorithm to an improving multipath model, avoiding the ambiguity between DOA and Doppler and increasing the estimation accuracy. Simulation results verify the superiority of the proposed algorithm in different multipath scenes and different incident angles. In the future, we will research on the optimization of algorithm complexity.
